# Efficient E-Mail Spam Detection Strategy Using Genetic Decision Tree Processing with NLP Features

**DOI:** 10.1155/2022/7710005

**Published:** 2022-03-24

**Authors:** Safaa S. I. Ismail, Romany F. Mansour, Rasha M. Abd El-Aziz, Ahmed I. Taloba

**Affiliations:** ^1^Department of Mathematics, Faculty of Science, New Valley University, El-Kharga 72511, Egypt; ^2^Computer Science Department, Faculty of Computers and Information, Assiut University, Assiut, Egypt; ^3^Information System Department, Faculty of Computers and Information, Assiut University, Assiut, Egypt

## Abstract

In this modern era, each and everything is computerized, and everyone has their own smart gadgets to communicate with others around the globe without any range limitations. Most of the communication pathways belong to smart applications, call options in smartphones, and other multiple ways, but e-mail communication is considered the main professional communication pathway, which allows business people as well as commercial and noncommercial organizations to communicate with one another or globally share some important official documents and reports. This global pathway attracts many attackers and intruders to do a scam with such innovations; in particular, the intruders generate false messages with some attractive contents and post them as e-mails to global users. This kind of unnecessary and not needed advertisement or threatening mails is considered as spam mails, which usually contain advertisements, promotions of a concern or institution, and so on. These mails are also considered or called junk mails, which will be reflected as the same category. In general, e-mails are the usual way of message delivery for business oriented as well as any official needs, but in some cases there is a necessity of transferring some voice instructions or messages to the destination via the same e-mail pathway. These kinds of voice-oriented e-mail accessing are called voice mails. The voice mail is generally composed to deliver the speech aspect instructions or information to the receiver to do some particular tasks or convey some important messages to the receiver. A voice-mail-enabled system allows users to communicate with one another based on speech input which the sender can communicate to the receiver via voice conversations, which is used to deliver voice information to the recipient. These kinds of mails are usually generated using personal computers or laptops and exchanged via general e-mail pathway, or separate paid and nonpaid mail gateways are available to deal with certain mail transactions. The above-mentioned e-mail spam is considered in many past researches and attains some solutions, but in case of voice-based e-mail aspect, there will be no options to manage such kind of security parameters. In this paper, a hybrid data processing mechanism is handled with respect to both text-enabled and voice-enabled e-mails, which is called Genetic Decision Tree Processing with Natural Language Processing (GDTPNLP). This proposed approach provides a way of identifying the e-mail spam in both textual e-mails and speech-enabled e-mails. The proposed approach of GDTPNLP provides higher spam detection rate in terms of text extraction speed, performance, cost efficiency, and accuracy. These all will be explained in detail with graphical output views in the Results and Discussion.

## 1. Introduction

Spam messages are the main issue that needs to be avoided or eliminated from the present scenario, because these kinds of spam mails affect the general mails with harmful events created. Because of such issues, it is an urgent need to identify that kind of harmful spam mails and avoid the harm from the present digital world. In the past era, there were several mail spam detection methodologies such as blacklist, whitelist, content-based data filter, and some machine learning based data identification strategies. However, all fell down under certain scenarios, and there is no existing method to provide a hundred percent spam detection ability to the present spam e-mail filtering system. The e-mail blacklist mechanism is nothing but a data store, which is able to store multiple spam e-mail IDs and provide support to users to detect whether the messages are coming from those stored mail IDs or not. If any mail is coming from the marked mail ID, the blacklist-enabled system immediately identifies that and reports it to the recipient. In some advanced systems, the IP-based data and mail filtering is available, which blocks the messages coming from suspected IP addresses and provides security to the present system in decent manner. The whitelisted e-mails are considered to be the known identity e-mails, in which the whitelist provides the ability to the user to block the known mail IDs and protect the system from the further coming mails. The particular whitelist-enabled mail protection system is also a data store, which stores the represented known-party mail IDs and provides security to the e-mail handling system in an efficient manner. The next variation of existing protection scheme follows content-based data filtering method, in which the received e-mail content will be extracted and filtered with the available block listed content, and if the content matches the block listed content, that mail will immediately be marked as spam or junk.

Recently, the massive Internet usage and the cost efficiency of e-mails attracted the advertisers for their marketing. Hence, the amount of received spam messages goes on increasing. Most of the advertisements are related to suspicious, “get rich faster with no efforts” subjects that attract the users and induce them to click on them and visit sites. Moreover, these spam messages are annoying for users due to not only spreading the spam contents, but also wasting the network bandwidth and taking more time in differentiating between the spam and legitimate messages, which can also dissatisfy the recipients and lead to damage to their systems with viruses and malware [1]. The amount of e-data like e-books, digital libraries, e-mails, e-newspapers, and e-publications is increasing rapidly, and hence it is more challenging to manage such electronic data. Text mining is a method of extracting the useful information from texts and also works on summarizing, classifying, and retrieving data. The major task of text mining approach is text classification (TC), also called text categorization. TC is a method of assigning text documents to predefined categories. Classification of a lot of text documents manually is a time consuming and expensive task. Therefore, research on automatic text management is vital in text mining [2]. Principal Component Analysis (PCA) is a technique based on biometric algorithm. PCA is a statistical analysis which makes use of orthogonal transformation for converting the correlated variables to uncorrelated variables. The PCA compresses multidimensional data at the time of retainment and also covers the techniques called covariance, standard deviation, and eigenvectors [3].

The problems caused by spam are solved by filtering them from the e-mails. This can be done by 3 approaches: simple, intelligent, and hybrid approaches. The simple approach includes munging, listing, aliasing, and challenging. The intelligent approach includes Artificial Neural Network (ANN), Support Vector Machine (SVM), Naïve Bayes, DNA Computing, and Artificial Immune System. The hybrid approach includes the combination of 2 or more methods so as to facilitate the improvement of performance and to overcome the issues faced by the single approach [4]. There are various techniques available to overcome the threats caused by spam e-mails. An antispam law is enacted with legislating penalty for e-mail spammers. Furthermore, it is believed that there are 2 approaches generally used in the detection of spam e-mails, namely, knowledge engineering and machine learning. Knowledge engineering enables the users to access the network information and IP address to differentiate the spam e-mails from original e-mails, which is called the origin-based filter. To distinguish between the spam and legitimate e-mails, a set of rules is to be framed in knowledge engineering approach. These rules must be framed by analyzing the filter techniques or by some authorized personnel. The second approach called machine learning is believed to be more effective and efficient than the approach of knowledge engineering, since the machine learning approach does not need any set of rules. The machine learning based spam filtering technique uses a set of preclassified e-mails which acts as a training dataset; then, with the help of some machine learning algorithms, the machine gets trained by the training sample, and finally a spam detection model is developed. Filtering is the common method for differentiating between the spam and nonspam messages [5]. Data mining is a method of integrating various techniques to analyze larger amount of data. These techniques include machine learning, artificial intelligence, pattern recognition, statistics, and database systems. Moreover, in the area of data mining, there are a lot of algorithms available to perform different types of tasks [6].

Text categorization is a method of automatic categorization of text documents into a set of predefined categories. The usage of digital documents has been increasing for the last few years. Thus, the text categorization technique is challenging. The feature extraction technique needs much focus in increasing the overall accuracy of a text classifier system, since text classification involves a large number of features including noisy, redundant, and irrelevant features, which results in overfitting in many classifier systems [7]. The spam e-mails mostly aim at stealing money from the recipients. Hence, spammers often offer products related to getting rich in few days; becoming a great business man without investments; getting miraculous healthcare that cures hair fall, obesity, diabetes, joint pains; getting instant glow beauty products; etc. The spammers have chosen these kinds of spam contents due to the fact that humans are considered to be naturally greedy. Some of the spam e-mails have sexual contents that redirect to pornography or some low-cost drugs that induces sex capability. Some countries enacted CAN-SPAM Act, which provides a set of rules about sending commercial e-mails; and if these rules are violated, this leads to penal actions. However, these countries also experience spam e-mails since the law is not implemented effectively [8]. Due to the popularity of the Internet, the electronic mail becomes the most widespread communication service all over the world, due to being cost-free. However, this feature is an added advantage of spammers to send bulk spam e-mails to earn money, steal personal information or online account details, or damage systems of the recipients by viruses or malware. This can be done due to the lack of trusted identity and security system in the current e-mail system which uses the Simple Mail Transfer Protocol (SMTP) that does not verify the origin of the e-mails. Currently available SMTP is open for abuse, because anyone can fake and send any content via e-mails called spam. Every day, a user is getting a certain number of spam e-mails from anonymous senders in their mailbox [9].

The elementary acoustic segmentation of speech is taken into account by the classification of speech into voiced, unvoiced, and silence. In addition to phonemes, the acoustic segmentation of speech is nearly identical to each alphabet's sound of which the human speech is comprised. In digital world, most of the contents can be readable and understandable by only a few who know a scrupulous language. The linguistic technology helps us in providing solutions in regular interface format so that this information can be readable by many of the people who speak various languages, especially in India which has a multilingual society with around 1652 dialects [10]. The Internet consists of infinite data repository with many distinct data domains like education, medicines, politics, and sports. The data in the domain are divided into many categories. The subspaces of data within the domain are processed as independent objects [11]. Researchers make use of web as a source of collecting information. The authors in computer literate field register millions of domains and publish billions of files over the web every day. Recently, the Really Simple Syndication (RSS) feeds are organized to provide us with time-based news for promoting viewership. These RSS feeds have information for researchers for their future researches [12].

Data mining is carried out using 2 methodologies, namely, (1) classification which is a supervised machine learning approach and (2) clustering which is an unsupervised learning approach. The data mining efficiently classifies the text data into several categories using algorithms. This classification involves several stages. The first is that the categories individually comprise of a set of rules based on the texts and classification problem. The final stage is using classification algorithms for predicting the category of the test document with the help of manually trained data [13, 14]. There is a rapid growth of digital products and services as a mediator for daily activities of humans. Hence, the use of e-mails in communication is also getting increased, which increases the number of unsolicited e-mails recently and causes serious threats to global economy and security. Since the e-mail communication is easier to access with minimum security and verification for creating new accounts, this technology is being misused to acquire the users' personal and sensitive data without their permission. Cybercrimes include trapping the confidential data of a user like account numbers, social security numbers, and passwords, which is called identity theft and is considered to be the most profitable crime carried out often by identity theft criminals. These unsolicited e-mails are masked as if they are received from a genuine web source, attract the recipients for redirecting them to fraudulent websites, and influence them to reveal or enter their personal information. The Kaspersky Lab reported that in 2019, first quarter, the threats caused by the unsolicited e-mails used 55.97% of traffic, which is 0.07% more than the percentage in 2018, fourth quarter. The unsolicited e-mails are classified as spam and phishing e-mails [15].

The phishing has been more refined now. Several antispam solutions have been proposed, and some are being implemented. However, these solutions do not provide good results, since every day more and more mails have to be dealt with by them. However, spam filters are the probable solution for filtering spam e-mails, even though they require Internet processing cost and use CPU and processing time. The technique called spam filtering excludes the unsolicited e-mails before being received into one's inbox. This technique is much popular, since it can do a lot of work related to eliminating unwanted e-mails. The spam filter works in such a way that the sensitivity of this filter can be controlled by the user, determining the criteria based on which the e-mails are to be filtered. It can be based on attachments, type of data, certain words, and much more. To build a safe and robust spam filtering model, a combination of multiple scanning methods is needed [16]. E-mails are the easy yet efficient communication medium for business and also for individuals to send and receive useful and confidential information. However, nowadays, spamming the e-mail messages is increasing by sending spoofing, phishing, and junk e-mails 60%‐70%, which is tedious and can be filtered from about 50 e-mails per day. These spam e-mails are sent to a user with the motive of creating more network traffic, hacking the user's account credentials, and wasting the time and energy of someone. The hackers have many types of codes for hacking or blocking anyone's account or modifying the contents in the account [17]. The problem in the existing spam e-mail filtering is its accuracy, which causes errors. There are several algorithms in machine learning that can be applied to e-mail spam filtering, and the most common among them is the Naïve Bayes algorithm due to its simplicity and implementation outcomes like less training time and faster evaluation time. The training for Naïve Bayes algorithm requires a set of spam e-mail and nonspam e-mail contents. The words in spam, nonspam, and both contents are tracked and used in the training process. This algorithm is used in many distinct datasets with distinct attributes and features [18].

The Internet traffic occurring nowadays is mostly due to the bulk spam e-mails. These spam e-mails needed proper measures to overcome the worse situation caused by them that ultimately weakens the e-mail usability. The e-mail IDs are collected by the spammers from websites, news groups, chat rooms, customer lists, viruses, etc. that capture the personal details of an individual and sell that information to the spammers. The challenges faced in filtering the spam e-mails are due to the large number of e-mails sent and received every day and the large number of features in datasets [19]. While analyzing the data published in 1998 regarding spam, it is found that the spam e-mails acquire an exponential growth in threatening attacks. A recent survey reported that the number of spam e-mails in March 2013 was around 100 billion, which is about 98% more than that in the previous quarter. The spam e-mails affect the production and finance of an organization leading to loss in productivity and economy. To eliminate this threat in Internet world, anti-spammers are taking several control measures. The spam filtering methods based on supervised machine learning are commonly used, making use of either header- or content-based features. Both of them are very simple to bypass, especially the header-based feature [20].

The proposed method of spam e-mail detection (GDTPNLP) is executed using Genetic Algorithm (GA) and Natural Language Processing (NLP), and the outcome is compared with the base models like Naïve Bayes, Support Vector Machine (SVM), Nearest Neighbor, and J48 to analyze the performance of the proposed model. The rest of this paper is organized as follows: Section 2 describes related works from the literature.  Section 3 briefly explains the concept of the proposed system.  Section 4 presents the performance measures applied over this paper.  Section 5 provides the results and discussion in clear manner. The last section, Section 6, provides the clear conclusion of the paper and the advantages of the proposed method, with proper future work discussion.

## 2. Related Works

A method for spam e-mail message filtering based on hybrid schemes is proposed in [1]. In that paper, the authors provide clear discussion regarding the advantages of e-mail spam filtering and the necessity of such systems in the current hacking-surrounded world. The authors also emphasize the necessity of spam-filter-enabled mail handling system in the face of the increasing amount of junk created for destructive uses. A machine learning strategy is presented in this paper, which is intended to provide a good spam identification process by means of systematic text extraction process and separate the legal messages or mails from the mailbox. A hybrid data protection scheme is introduced, in which the concepts of Complementary Naïve Bayes (CNB) and Multinomial Naïve Bayes are integrated together to provide high accuracy results. Along with these machine learning strategies, some of classification schemes are also used in this approach such Support Vector Machine classifier and Random Forest classification strategies to attain good classification accuracy with reduced error rates in outcome. The main benefit of this paper is that it attains an accuracy level of 99% in the optimization of results. In [2], text classification principles in association with genetic procedures and decision tree process with respect to parametric state finer are proposed. In this paper, the most important data mining role called text mining is applied to the proposed approach to perform text categorization for identifying the junk. This paper applied decision tree approach to categorize the text into unique content based on the trained samples, in which it is easy to predict the negative terms with respect to the prediction database. This paper empirically analyzes the process of text classification in more detail compare to the existing system with more precision. A high-standard fitness principle is applied to identify the junk presented in the text given by the user for testing. The trained data samples are categorized into several strategies with proper confidence-threshold levels. Once the given testing sample crosses the value of the mentioned trained text confidence level threshold, it will be marked as a junk. This paper principally classifies the input processing text based on three different variances, namely, True Positive, True Negative, and False Negative. In [3], Principal Component Analysis is proposed, and the authors described more details regarding PCA with respect to biometric data protection strategy. This paper insight specialized and utilized symmetrical change to change over a lot of perceptions of perhaps associated factors into a lot of estimations of directly uncorrelated factors. Likewise, Principal Component Analysis is a system used to lessen multidimensional information to bring down measurements while holding a large portion of the data. It covers several common deviation measures, covariance, and eigenvector calculations. This foundation information is intended to make the Principal Component Analysis area direct, yet it can be skipped if the ideas are as of now recognizable.

An intelligent method of detecting e-mail spam using Incremental SVM and Artificial Immune System is proposed in [4]. In this method, the several classifiers that classify the e-mails independently are held in a window, and the labels for the e-mails are provided using majority voting scheme. In the window, each classifier is dynamically updated using a method called exceeding margin update. To eliminate the outdated knowledge, a sliding window is used. These techniques provide an adaptive and dynamic property to track changes in e-mail contents and user interests periodically. PU1 and Ling public benchmarks are used to experiment with the above techniques, and the results showed that this intelligent spam detection model performs promisingly [4]. In [5], a method regarding hybrid data evaluation scheme is proposed for identifying e-mail spam. In this paper, hybrid data processing logic is proposed, which is an integration of negative-acquiring process and differential calculations with respect to artificial intelligence strategy. This paper concentrates more on the process of identifying the normal and abnormal users over the web medium and provides the hybrid mechanism to prevent those abnormal user activities over the environment. In this paper, the authors employed many research mechanisms described earlier to process such e-mail-based filtering process, but all are stuck in certain way of protection. That is the reason why a hybrid scheme is introduced in this paper to provide decent security norms to the e-mail pathway with respect to differential calculations and the negative-acquiring process. This paper adapts the nature of two variant algorithms such as negative-acquiring process and differential calculations to provide a maximum accuracy of 83.06 percent which is cross-verified with some other classical data protection and extraction algorithms. In [6], the authors use the top 10 data mining algorithms that are more influential in research community, which were suggested by the IEEE International Conference on Data Mining held in Dec 2006. They are KNN, K-Means Clustering, Apriori, SVM, Naïve Bayes, AdaBoost, EM, CART, PageRank, and C4.5. This paper describes each of the algorithms, its impacts, and a review of its current and future researches. The algorithms perform most of the crucial steps in the research and development of data mining like classification, link mining, association analysis, statistical learning, and clustering.

An overview of Principal Component Analysis (PCA) is given in [7], where the PCA is used for feature extraction using different classifiers. After the use of PCA, the classifier performance is monitored, and it is found that the data dimension is reduced effectively. This system is examined using 3 UCI datasets namely, DBWorld e-mails, CNAE-9, and Classic03, for classification performance using various popular text classifiers. The results showed that the use of PCA enhanced the performance of most classifiers. In [8], a process related to e-mail spam identification by using junk-mail filtering strategies is proposed. This paper states that junk mail is the major issue in the digital data processing industry. Generally, all commercial and noncommercial organizations trying to promote their products and new gadgets via e-mails, and all of them are taught that the e-mail is the best way of easy marketing. This concern attracts the intruders to do several harmful processes by sending spam associated e-mails and trying to affect the messages by default. The authors illustrated the work of several researchers, analyzed many solutions to the spam detection principle, and provided optimal solutions to eliminate junk process, but the content effect still remains in this era. For this reason, this paper introduced a hybrid data filtering scheme, provided the best solution to identify the junk mails and eliminate them from the scenario, and provided high level of data handling accuracy in the results. The entire paper provides practical proofs and outcomes to show the accuracy of the proposed hybrid data filtering strategy in avoiding the junk mails. In [9], a methodology combining machine learning and predictive analysis and working on cloud is proposed. A prototype is developed for the predictive analysis, which works on Microsoft Azure with the help of Azure Machine Learning technique for creating and deploying behavior models of e-mail server.

An overview of the perspective and obligation of the advancement of converting speech to text and the steps involved in each stage of classification process are described in [10]. For each stage, a comparative analysis is performed, and it is concluded that a system for future computing systems should be developed, with the language being mother tongue for speech recognition. The paper attempted to develop and implement an online system with Human-Computer Interface in mother tongue for speech recognition process and speech-to-text conversion. Moreover, modern algorithms and methodologies are used to process the speech signals for recognition into text format. The author also suggests that the real-time speech-to-text conversion in usual written language needed specific techniques, since this conversion must be very fast and should be accurate for almost 100% for understanding. A popular test bench for examining the text categorization techniques is the Reuters Corpus. A hierarchical organization is presented in the Reuters Corpus for testing the performance of classification of a hybrid architecture, with 4 major groups. Here, the headlines of Reuters Corpus are used, since it provides a short summary of each article. A single Reuters Corpus headline includes a single line of text data with 3 to 12 words [11]. For text classification in documents, a general and special case of a Hy-RNC is explored due to its potential in TC. In general case Hy-RNC, an ensemble Simple Recurrent Network (SRN) is used with a boosting unit that consists of AdaBoost algorithm named NeuroBoost, which is operated based on the output from networks of hybrid neural classification, in addition to extraction of weak hypotheses in output. In special cases, a single SRN is trained and the data is passed directly to the boosting unit and then given to the hybrid classifier via NeuroBoost algorithm [12].

A combination of Maximum Entropy and Naïve Bayes classifiers is used for TC in [13]. The Naïve Bayes method is a simple method of classification, but the method of Maximum Entropy supports much uniformity and flexibility. Both have distinct assumption models. The assumption model for Naïve Bayes includes complete independency of the words in a text document, which is considered as impossible, whereas the Maximum Entropy is approximately related to the realistic cases. There have been different available efficient modification techniques for tradition Maximum Entropy classifier, and the modified classifier and the Naïve Bayes classifier are combined with the help of 3 operators for merging, viz., average, max, and harmonic mean. The performance of this system is analyzed, and it is concluded that none of the classifiers individually achieves much better performance. An application that uses Blitzer's dataset for text analysis is proposed in [14]. The classifiers used in this application are Naïve Bayes and Maximum Entropy as seen in [13]; and the results are combined using merging operations like max, average, and mean; and better overall performance than that of the individual classifier is achieved. In [15], a tutorial for the scientists and security analysts who need the benefits form the data in existing e-mails is presented. Hence, at first the important features form e-mail corpus are extracted, and with the help of 6 feature selection techniques, the optimal feature subspace that provides an efficient learnability of machine learning models is determined to impact the UBE predictability. The feature selection techniques are Principal Component Analysis, Feature Importance-Based Filtering, Variance-Based Filtering, Minimum Redundancy, Maximum Relevance, and Correlation-Based Filtering. At last, the extracted optimal feature subspace is evaluated using 8 machine learning algorithms, namely, Naïve Bayes, Random Forest, Bagged Decision Trees, AdaBoost, Support Vector Machine, Voting Ensemble, Stochastic Gradient Boosting, and Extra Trees, and achieves an overall classification performance of 99% of UBEs [15].

A Python based spam filtering model is described in [16], in which the interesting spam or ham words are extracted from the training dataset at first, and then with the help of this spam-ham lexicon, training and testing tables that can be used by various algorithms of data mining are generated. This model is finally evaluated with a dataset, and it is found that the results of this spam filtering approach impact the performance of SVM and Naïve Bayes classifiers. An integrated model of spam e-mail detection with machine learning based Naïve Bayes (NB) algorithm and computational intelligence based Particle Swarm Optimization (PSO) algorithm is proposed in [17]. The NB algorithm learns and classifies the spam and nonspam e-mail contents. The PSO algorithm contains swarm behavior and stochastic distribution properties used for global optimization of parameters in NB algorithm. This proposed model is examined with Lang spam dataset; the performance evaluation is done based on precision, accuracy, recall, and F-measure; and the results show that the PSO performs better than the individual Naïve Bayes algorithm. In [18], e-mail spam filtering based on Naïve Bayes approach is presented, and its performance is tested on 2 datasets, namely, SPAMBASE and Spam Data datasets. The performance measures implemented in this approach are the precision, accuracy, recall, and F-measure. WEKA tool is used here for evaluating the NB based spam e-mail filtering using both the datasets, and the acquired results showed that the performance of NB approach varies with the e-mail type and the number of dataset instances.

A Support Vector Machine based spam classification method is proposed in [19]. The SVM classifier is a powerful yet nonlinear classification approach that can be used for complex problems of classification. An efficient Ant Colony Optimization (ACO) approach is used for feature selection technique. The performance evaluation in this method is done by calculating its accuracy, and the results show good performance. A method of spam classification based on features from the combination of e-mail content language and readability with existing content-based task features is proposed in [20–22]. The feature extraction is carried out using 4 benchmark datasets, namely, SpamAssassin, CSDMC2010, Enron-Spam, and Ling-Spam. For implementing this proposed spam filter, 5 popular algorithms are used, namely, Naïve Bayes, Support Vector Machine, AdaBoostM1, Bagging, and Random Forest approach. After the evaluation of the above-mentioned classifiers, it was found that Bagging outperforms the remaining algorithms and also the approaches in the existing researches. A limitation of this approach is that it is implemented only in English language. However, it is assumed that it works well in classification of spam e-mails in other languages too.

The above survey provides a clear understanding and basic and advanced knowledge regarding the spam e-mail filtering and classification process and the algorithms and techniques used for attaining the desired outcome and the performance evaluation measures employed in the existing studies [23–25].

## 3. Proposed System Methodologies

The proposed methodology of e-mail spam detection scheme is completely different from existing approaches in terms of intelligence and accuracy. This paper proposed the system of Genetic Decision Tree Processing with Natural Language Processing (GDTPNLP) concentrating on dual end of data protection over mail platform: one is the textual mail conversation and the other one is the voice-enabled mail conversation. All the existing schemes for identifying the mail spam are suitable only for textual mail conversations, but the proposed system GDTPNLP supports protective mechanism for both voice and textual mail conversations. The proposed system adapts Natural Language Processing to provide support to the voice-enabled mailing systems, which is illustrated in [10], where the authors described the advancements of such voice-enabled systems; however, in this paper, our proposed algorithm called Genetic Decision Tree Processing with Natural Language Processing provides complete protection from spam and intruders. This protection guard gives more accuracy to our proposed system which will be clearly illustrated in the Results and Discussion. The following summaries will illustrate the proposed approach logic in detail, which is the hybrid logic and belongs to two different algorithms as well as including the benefits of both the algorithms in the proposed approach. These all will be further described one by one in detail.

### 3.1. Dataset Description

The textual dataset is considered here for training the proposed approach, and the resulting accuracy levels are denoted based on such training samples. The set of preclassified e-mails acts as a training dataset. The dataset undergoes a preprocessing step for removing the stop words and POS tagging for extracting the nouns, verbs, and other needed criteria.

**Figure 1:**
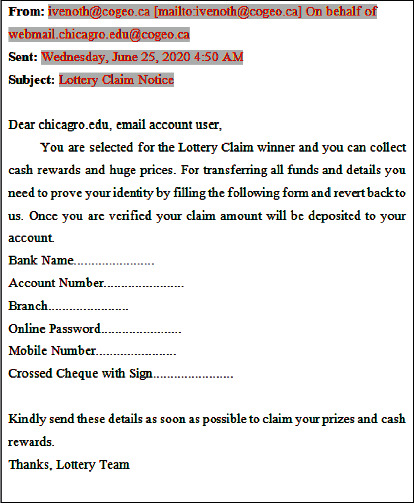
Sample spam e-mail.

Nowadays, a huge volume of attractive spam and infectious mails arrives from multiple attackers or intruders. For example, consider Figure 1, which illustrates the received sample spam e-mail, which requests several personal and unwanted information regarding bank-account details of an individual.

**Figure 2:**
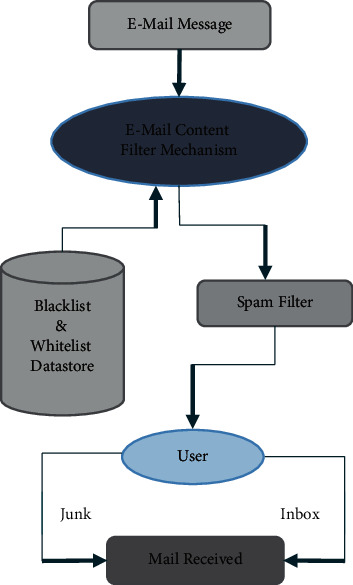
E-mail spam filter procedure model.

Figure 2 illustrates the model view of spam filter work and the process flow in detail. This system of content-based data extraction policy of the e-mail system is considered to be the probabilistic approach, which identifies the blocked content based on probability. If the content is used for positive sentence, the system cannot identify that. Thus, this method is also considered to be noneffective data and e-mail protection scheme. Therefore, some advanced e-mail security handling system is required to provide protection to the present e-mail processing. That is the reason why, in existing approaches, some machine learning schemes are also introduced based on data classification principle. The machine learning systems are usually trained with some predefined dataset variations and process the present data with the existing trained data. If the testing (present input) data matched the existing trained data, that will immediately be noticed by the e-mail protection system and further steps of blockings will be taken accordingly. Numerous researchers tried with many algorithms to provide protection to the e-mail systems based on such text extraction strategies. The success level of such systems is acceptable; specifically, those systems of machine learning principles provide efficient results with good precision rate. However, these methodologies suffer some lack of identification of spam e-mails based on text categorization and need some more training, and heavy data store relevant to multiple keywords is required to prove the efficiency of machine learning principles. In general, multiple classification strategies are already available, such as Support Vector Machine, K-means, and Naïve Bayes classification principles.

This is only the case of text messages over mail, we are talking about, but in case of voice mails, the complexity is a bit increasing, and the protection rate is to be enhanced based on that. This voice-enabled mail pathway requires voice-to-text conversion strategies to convert the voice to text and process the text based on the methodology applied. In the proposed system, a new methodology is required to provide good support to identify the spam e-mails with high accuracy and prediction rate, which is called Genetic Decision Tree Processing with Natural Language Processing (GDTPNLP), which is nothing but a hybrid algorithm. The term hybrid itself says it is an integration of two different algorithms, namely, Genetic Algorithm and Decision Tree Processing. Along with this proposed principle, a feature extraction strategy called Principal Component Analysis (PCA) is also applied to improve the accuracy rate of the proposed system and provide high-precision rate with low-processing overhead. This PCA eliminates the processing time of the server and provides a high accuracy outcome.

### 3.2. Genetic Procedure

**Figure 3:**
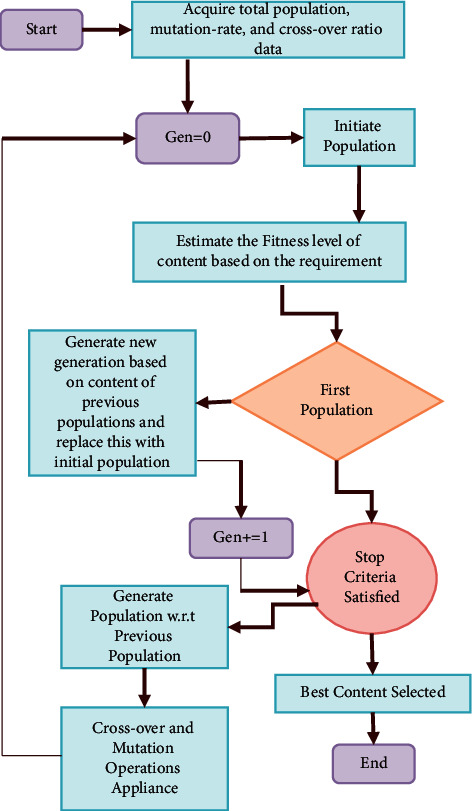
Genetic procedure: schematic structure.

The genetic procedure is favored in the proposed approach because of its genetic and natural processing stability, as shown in Algorithm 1. By applying this genetic procedure, the signs of past research implementations can easily be cross-checked with the proposed system; in addition, this kind of genetic process is followed to attain high accuracy solution to optimization and data-search procedures with respect to the bioinspired processing like “mutation,” “crossover,” and “selection.” Figure 3 illustrates the genetic procedure in detail.

The Genetic Algorithm (GA) is an evolutionary algorithm which is based on the Darwinian natural selection. It selects the individual that is a best fit in the given population during iteration based on the principles of inheritance, variation, and selection. The GA has maintained a fixed size of population with the unique number of individuals which is represented in binary. As fitness increases, the probability of reproduction of offspring also increases. This program is executed and run for 10 generations with a population size of 40; i.e., configuration of 400 (10 × 40) hyperparameters is evaluated. Each pipeline is evaluated for 10-fold cross validation, i.e., 400 × 10. The completed pipeline is calculated using

(1) $$\begin{array}{c} \left(\text{Generation }*\text{ lambda}\right)+\text{population size}=240\text{ times,} \end{array}$$

where lambda is the offspring size.

Without offspring size, the pipeline can be evaluated by using the value of population size in the place of lambda, and the mutation rate (0.9) and crossover rate (0.1) are default values. It must be ensured that the sum of mutation and crossover rates should not exceed 1.0.

### 3.3. Decision Tree Processing

Decision Tree Processing is an important and efficient machine learning strategy, which is commonly used for classification task. The concept of Decision Tree Processing is simple to make and simple to adapt; furthermore, this process has complete performance improvements based on processing speed and efficiency. Thus, the decision tree process provides higher accuracy rates over resulting unit classification principles. This decision tree development does not need any domain information, it has the capacity for choosing the most unfair textual components from a colossal amount of raw text, and it can manage noisy information. Thus, utilizing decision tree is a decent decision for text information grouping with the end goal that text information is affected by a tremendous number of features. It is a flow-like tree structure where inside hubs signify a test on a property, each branch indicates a result of test, and the stems foresee the classification [16].

Finding the ideal size of the last tree is one of the most significant issues that emerge in decision tree, with the end goal that a tree that is too huge causes overfitting to the training data and ineffectively summing up to new examples. Additionally, little tree will not probably catch significant auxiliary data about the sample region. In Decision Tree Processing, pruning is another concept, which is used to eliminate the unimportant data in the given data for testing. The elimination of such data from the concerned processing gives a huge impact on data size and enhances the accuracy levels of the result. The overall complexity of classification process is reduced based on this pruning principle.

The decision tree (DT) is a method of predictive analysis. The decision tree works on the basis of creating a category which then creates its own subcategories, and each subcategory then creates its own subcategories. This process goes on until the program is being terminated by the programmer or at the time of reaching its desired outcome. The prediction of outcome depends on the training data which is used in the learning process. The longer and deeper the tree develops, the more complicated the rules are expected to be during execution. Algorithm 2 gives the pseudocode of decision tree.

### 3.4. Principal Component Analysis

The Principal Component Analysis (PCA) concept is an efficient factor, which is used to extract the analytical features from the input data, and it can decrease the high-volume dataset, which consists of huge-noise data values. This kind of noise data occupies more space of the dataset, creates processing confusion, and delays the entire process and its performance. The main concentration of Principal Component Analysis structure is to create a subset of information from the huge dataset with respect to the original operator and operands associated with it. The correlation of such subsets is called principal component and which is more or less equal to the actual dataset features. This Principal Component Analysis is used in the proposed approach to eliminate the high-dimensional features of the input data to improve the efficiency and robustness of the proposed system and its functions.

### 3.5. Data Preprocessing

Data preprocessing is the most important methodology in data and feature extraction process, which is easily adaptable in machine learning procedure. This data preprocessing procedure is acquired in the proposed approach to efficiently identify the spam mails. Data preprocessing is the machine learning procedure, especially used to extract the data from the given dataset and process the data according to the requirements. The major concern of this data preprocessing stage is to format the standard structure of outgoing mails by using text classification principles. The formal steps carried out over this process are as follows:

- Humans and computers interact.
- Eliminate the HTML tags.
- Extracted contents from the mail body are tokenized uniformly.
- Remove the words with length less than or equal to two.
- Remove all the special characters of the word.
- Eliminate the stop words available into the message body, for example, pronouns and prepositions. These are all creating unwanted space occupancy and do not produce any meaningful sentence over processing.
- Perform stemming procedure, which is the most known procedure for returning the words to the tree roots.

Once the preprocessing process completed, the processed and retained words are covered over the cluster as well as forming the unique identity of that, so that the classification principle can easily accumulate and process the same.

### 3.6. Feature Weight Analysis

In this progression, the mail messages are encoded into a mathematical portrayal that text classification principle can deal with. One of the most powerful, viable, and user-friendly plans for feature weighting is Term Frequency–Inverse Document Frequency (TF-IDF) calculation. In TF-IDF calculation, the weight for portraying the commitment to data substance of list section is utilized to be determined. Appropriately, let *TF*_*i*_ be the number of events of *T*_*i*_ in the archive and *dF*_*i*_ be the quantity of the report wherein the *T*_*i*_ term is seen at any rate once. The *IDF*_*i*_ is determined by the accompanying condition given in

(2) $$\begin{array}{c} IDF_{i}=\left(\frac{D_{y}}{dF_{i}}\right), \end{array}$$

where *D*_*x*_ indicates the total set of documents acquired from the training set, and the weight of every word is estimated by

(3) $$\begin{array}{c} WX_{i}=\left(TF_{i}.IDF_{i}\right). \end{array}$$

### 3.7. Natural Language Processing

A branch of artificial intelligence named Natural Language Processing (NLP) facilitates the communication between humans and computing systems with the help of natural languages. The natural language is the one which can be easily understood by the users by naturally reading and deciphering it. The main aim of NLP is to use the natural local languages spoken by the human beings valuably. The machine learning methods used in the execution of NLP are as follows:

- Interaction between humans and computers.
- Capturing audio by a device.
- Converting audio to text.
- Processing the text data.
- Converting data to audio.
- Producing the final outcome, the device playing the audio.

The aim of Natural Language Processing is to identify the rules of natural languages and extract them by converting the unstructured data into computer understandable data. This is done by extracting the text that is entered in the computer using certain algorithms and extracting only the mandatory information from it. It also has the limitation of not efficient understanding of some text data, and this might cause obscured outcome. The techniques involved in the process of NLP are as follows:

 
**Lemmatization:** lemmatization is the process of reducing the words with various modified forms into a single form.
 
**Morphological segmentation:** morphological segmentation means dividing the words into units named morphemes.
 
**Word segmentation:** word segmentation is the process of dividing the larger continuous text into various units.
 
**Part of speech tagging:** part of speech tagging is the process of finding the parts of speech of each of the words.
 
**Parsing:** parsing is the process of analyzing the syntax and grammar of each sentence.
 
**Sentence breaking**: sentence breaking is the process of breaking the sentences of a larger text by placing boundaries.
 
**Stemming:** stemming is the process of cutting modulated words into their root form.

Algorithm 3 gives the pseudocode for NLP approach.

### 3.8. Genetic Decision Tree Processing with Natural Language Processing (GDTPNLP)

The proposed hybrid algorithm of Genetic Decision Tree Processing with Natural Language Processing (GDTPNLP) integrates the benefits of both the genetic procedure and the decision tree progression. This proposed algorithm is bidirectionally used for both textual and voice-assisted e-mails sent from one end to another end. The genetic procedure extracts the content from testing input or live composed mail and cross-checks it with available trained set in a genetic manner. The decision tree process is used for classifying those results in an intelligent manner and provides the estimated result of the present mail message as a junk or normal mail. The proposed algorithm of GDTPNLP assigns the confidence-threshold level to every mail message and then cross-checks the message with trained set; if the trained set value exceeds the present message threshold level, then the proposed algorithm marks this mail as junk, and a similar process is followed for voice mail messages as well. However, the concept of data extraction is a little bit different for voice mail message extraction principle. The voice data is collected from the user via Google Assistant, and the collected voice is extracted as a text message by using speech synthesizer. Once the speech variables are converted into textual form, the gathered input is assigned to preprocessing stage and processed further one by one similarly to text message analysis process. Finally, the Genetic Decision Tree Processing with Natural Language Processing procedure is applied to the input, and it analyzes the present voice message to determine whether it is proper or it contains any spam content. If the process identifies spam content in the input, then the mail is forwarded to the junk folder of the mailbox; otherwise, it will be forwarded to the inbox of the user. Algorithm 4 is given below in detail for GDTPNLP proposed algorithm.

## 4. Performance Measures

The dataset was obtained from the UCI Machine Learning Repository [26], and it has 58 attributes (57 continuous attributes and 1 nominal class label attribute), with a total of 4601 instances.  Table 1 shows the whole dataset along with a description of each attribute.

The various performance measures used in the proposed methodology are given as follows.

### 4.1. Confusion Matrix

With the help of various performance measures, it is possible to evaluate the spam e-mail detection. A Confusion Matrix is used to detect the performance for the spam e-mail detection model, as shown in Table 2.

True Negative Rate (TNR) is the ratio of True Negatives to sum of True Negatives and False Positives, as follows:

(4) $$\begin{array}{c} \text{TNR}=\frac{TN}{TN+FP}\;. \end{array}$$

True Positive Rate (TPR) is defined as the ratio of True Positives to sum of True Negatives and False Positives, as follows:

(5) $$\begin{array}{c} \text{TPR}=\frac{TP}{TP+FN}. \end{array}$$

False Negative Rate (FNR) is the ratio of False Negatives to sum of False Negatives and True Positives, as follows:

(6) $$\begin{array}{c} \text{FNR}=\frac{FN}{FN+TP}. \end{array}$$

False Positive Rate (FPR) is the ratio of False Positives to sum of False Positives and True Negatives, as follows:

(7) $$\begin{array}{c} \text{FPR}=\frac{FP}{FP+TN}. \end{array}$$

### 4.2. Accuracy

The accuracy of a model depends on the prediction of true values of spam and ham and is expressed as follows:

(8) $$\begin{array}{c} \text{Accuracy}=\frac{TP+TN}{TP+TN+FP+FN}*100. \end{array}$$

### 4.3. Recall

The recall is the measurement of the number of True Positive values predicted in a given set of spam e-mails as follows:

(9) $$\begin{array}{c} \text{Recall}=\frac{TP}{TP+FN}. \end{array}$$

### 4.4. Precision

The precision is the measurement of the number of true values predicted in a given set of positive e-mails as follows:

(10) $$\begin{array}{c} \text{Precision}=\frac{TP}{TP+FP}\;. \end{array}$$

### 4.5. F1-Score

The F-measure *F*_*β*_ is calculated by using precision and recall scores. The value of *F*_*β*_ gives the value of F1-score. F1-score is defined as the harmonic mean of precision and recall and is expressed as follows:

(11) $$\begin{array}{c} F_{\beta }=\frac{\left(1+\beta^{2}\right)\left(\text{Precision}*\text{Recall}\right)}{\left(\beta^{2}*\left(\text{Precision}+\text{Recall}\right)\right)}. \end{array}$$

With *ß* value = 1, the formula becomes

(12) $$\begin{array}{c} F_{\beta }=\frac{2*\left(\text{Precision}*\text{Recall}\right)}{1*\text{Precision}+\text{Recall}}. \end{array}$$

## 5. Results and Discussion

In the proposed approach, the results are empirically analyzed, and the proof of outcome is described below with graphical view in clear manner. The effectiveness of the proposed algorithm Genetic Decision Tree Processing with Natural Language Processing (GDTPNLP) is clearly analyzed by means of the above-mentioned summaries as well as the practical proofs in this section. Some of the classical data and text classification algorithms are listed in Table 3 to show the efficiency of the proposed system, which gives better accuracy and high tolerance rate compared to all the other existing approaches.

**Figure 4:**
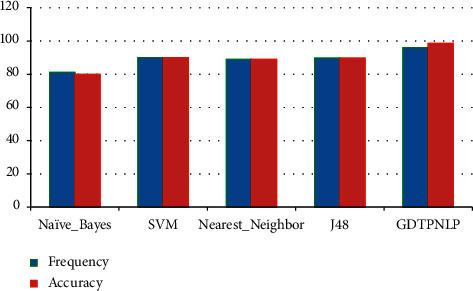
Proposed algorithm GDTPNLP accuracy and frequency comparison with different traditional algorithms.

Figure 4 illustrates the proposed algorithm frequency and accuracy level comparisons with multiple traditional approaches.

**Figure 5:**
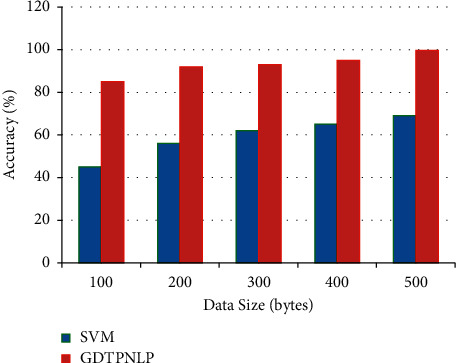
Graphical representation of textual feature extraction accuracy.

Figure 5 illustrates the textual feature extraction accuracy rate of the proposed system in order to compare it with the regular feature extraction process of the other classical approaches, as shown in Table 4.

**Figure 6:**
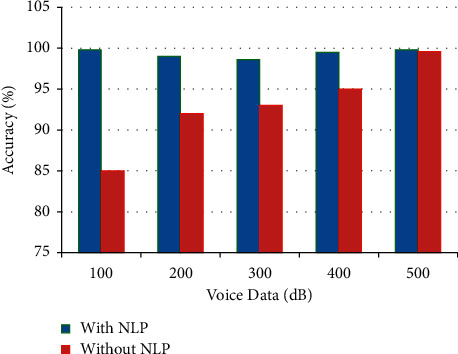
Graphical representation of speech-to-text conversion accuracy.

Figure 6 illustrates the speech-to-text data conversion accuracy level with and without Natural Language Processing from a classical view point, as shown in Table 5.

**Figure 7:**
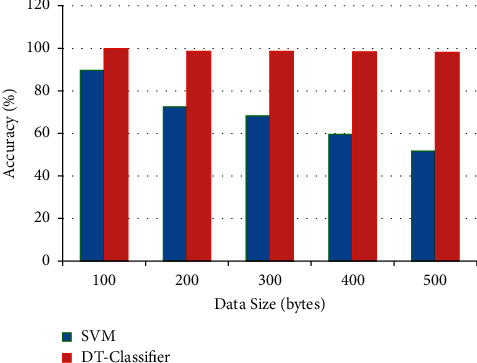
Graphical representation of classification accuracy.

Figure 7 illustrates the spam identification accuracy by using the decision tree classification principle as compared with the classical SVM classification algorithm, as shown in Table 6.

Table 7 shows computation time versus dataset size for the learning tasks in e-mail spam detection. The training costs are prohibitive for large-scale content-based spam detection or a large blog host.

**Figure 8:**
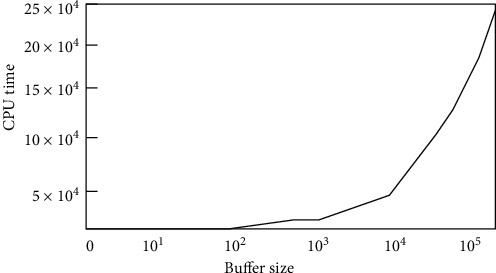
Buffer size vs. CPU time.

Figure 8 illustrates the effect of the buffer size in relationship to the CPU seconds required. The outcome indicates that the buffer size value less than 100 illustrates result with degraded performance, in spite of the very quick evaluation.

## 6. Conclusion and Future Scope

In this paper, an efficient e-mail spam detection methodology is introduced, called Genetic Decision Tree Processing with Natural Language Processing (GDTPNLP), which can support both textual and voice-assisted e-mail messages; in addition, internally, this algorithm is built with proper Natural Language Processing skill, so that the voice mail spam can easily be identified. The proposed algorithm GDTPNLP provides highly intelligent outcome with respect to classification accuracy, speech-to-text conversion accuracy, and so on, which is clearly illustrated in the Results and Discussion. The hybrid algorithm of GDTPNLP integrates both the features of genetic procedure and decision tree principles in association with Natural Language Processing feature as well. Hence, the textual as well as voice-oriented mails are easily identified with proper accuracy terms, and the outcome shows efficiency at each level of result. The Principal Component Analysis scheme dynamically extracts the features in order to reduce the data overhead in the classification process. By using the proposed GDTPNLP logic, the mail spam will be easily identified without any doubt. This work will be extended in the future based on the aspect of time efficiency, in which the processing time for data preprocessing and feature extraction principles need to be reduced further by applying some other optimization algorithms such as N-Complex, so that it will be more suitable for real-time applications in a good manner.

## Figures and Tables

**Algorithm 1:**
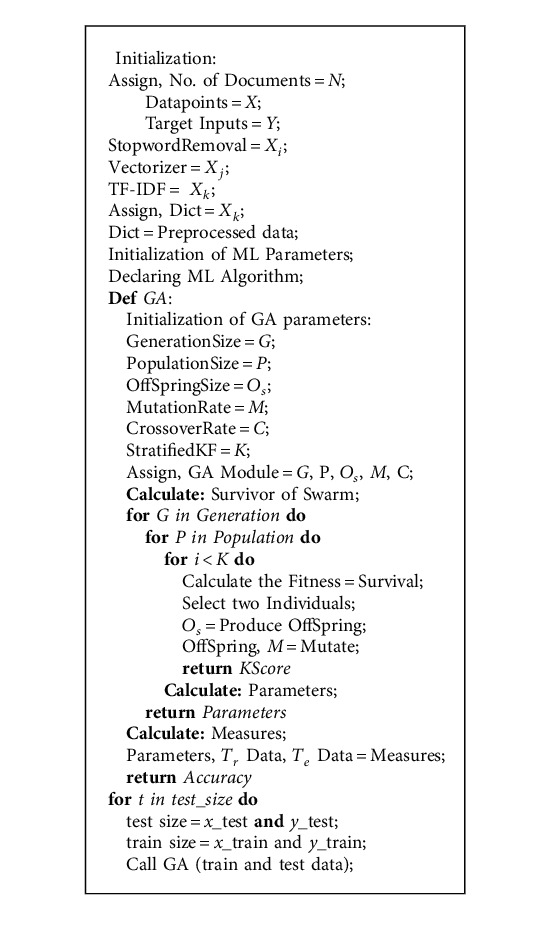
Genetic Algorithm.

**Algorithm 2:**
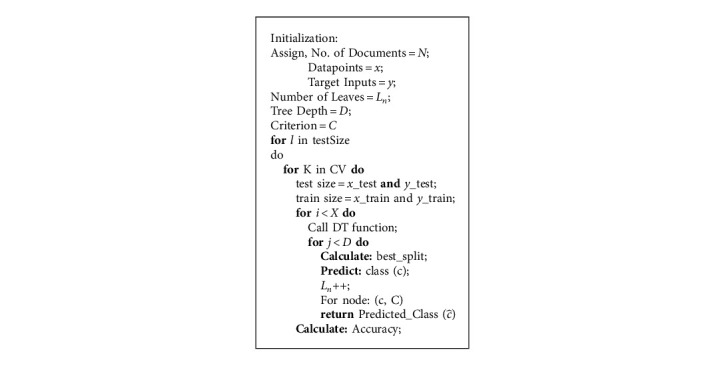
Decision tree.

**Algorithm 3:**
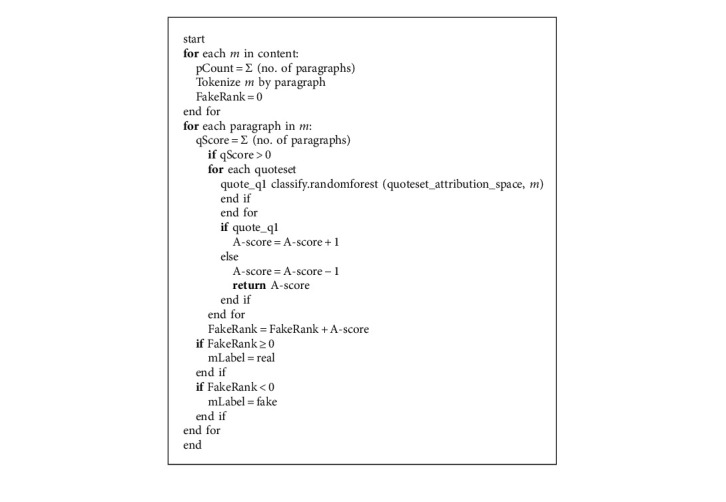
Natural Language Processing.

**Algorithm 4:**
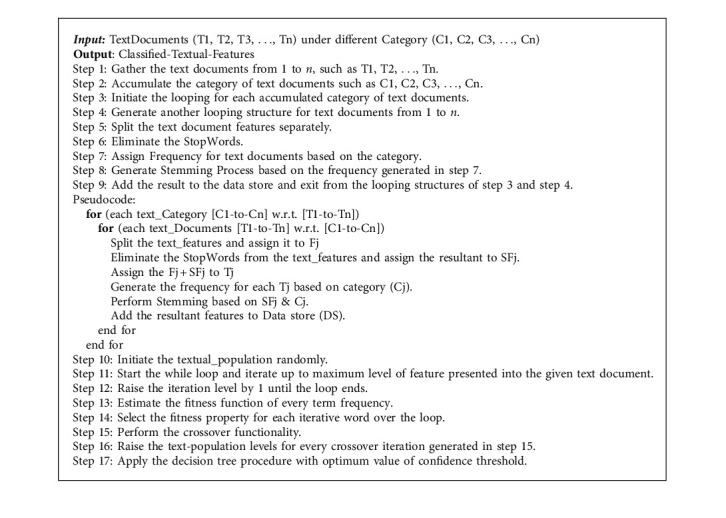
Genetic Decision Tree Processing with Natural Language Processing (GDTPNLP).

**Table 1:** Description of dataset attributes.

Attributes	Type	Description
1–48	char_freq_CHAR	The number of characters in an e-mail that are the same as CHAR.
49–54	capital_run_length_average	The average length of consecutive capital letter sequences
55	capital_run_length_longest	Longest consecutive capital letter sequence length
56	capital_run_length_longest	Longest consecutive capital letter sequence length
57	capital_run_length_total	Overall capital letters in e-mail
58	Class attribute	Indicating if an e-mail is classified as spam with class label (1) or not spam with class label (0)

**Table 2:** Confusion Matrix.

Metrics	Ham	Spam
Ham	TN	FP
Spam	FN	TP

Note. TN: True Negative (ham predicted as ham), TP: True Positive (spam predicted as spam), FP: False Positive (spam predicted as ham), FN: False Negative (ham predicted as spam).

**Table 3:** Proposed algorithm efficiency and frequency comparison with several traditional algorithms.

Algorithm	Frequency (%)	Accuracy (%)
Naïve Bayes	81	80
Support Vector Machine	90	90
Nearest Neighbor	89	89
J48	89.7	89.7
GDTPNLP	95.9	98.6

**Table 4:** Textual feature extraction accuracy.

Data size (bytes)	SVM	GDTPNLP
100	45	85
200	56	92
300	62	93
400	65	95
500	69	99.6

**Table 5:** Speech-to-text conversion accuracy.

Voice data (dB)	With NLP	Without NLP
100	99.8	85
200	99	92
300	98.6	93
400	99.5	95
500	99.8	99.6

**Table 6:** Classification accuracy.

Data size (bytes)	SVM	DT classifier
100	89.6	99.8
200	72.5	98.6
300	68.2	98.5
400	59.5	98.3
500	51.6	98.1

**Table 7:** CPU execution time for e-mail spam detection.

Features	Computation time (s)
Words	12196.1
3 grams	44605.3
4 grams	87519.42

## Data Availability

The data that support the findings of this study are available from the corresponding authors upon reasonable request.
